# Immunotherapy Vaccines for Prostate Cancer Treatment

**DOI:** 10.1002/cam4.70294

**Published:** 2024-10-28

**Authors:** Jide He, Jialong Wu, Ziang Li, Zhenkun Zhao, Lei Qiu, Xuehua Zhu, Zenan Liu, Haizhui Xia, Peng Hong, Jianling Yang, Ling Ni, Jian Lu

**Affiliations:** ^1^ Department of Urology Peking University Third Hospital Beijing China; ^2^ Institute of Medical Innovation and Research Peking University Third Hospital Beijing China; ^3^ Institute for Immunology and School of Medicine Tsinghua University, Medical Research Building Beijing China; ^4^ State Key Laboratory of Natural and Biomimetic Drugs Peking University

**Keywords:** immunotherapy, prognosis, prostate cancer, therapeutic tumor vaccine, tumor treatment

## Abstract

**Background:**

Therapeutic tumor vaccines have emerged as a compelling avenue for treating patients afflicted with advanced prostate cancer (PCa), particularly those experiencing biochemical relapse or ineligible for surgical intervention. This study serves to consolidate recent research findings on therapeutic vaccines targeting prostate tumors while delineating prevalent challenges within vaccine research and development.

**Methods:**

We searched electronic databases, including PubMed, Web of Science, Embase, and Scopus, up to August 31, 2024, using keywords such as 'vaccine', 'prostate cancer', 'immunotherapy', and others. We reviewed studies on various therapeutic vaccines, including dendritic cell‐based, antigen, nucleic acid, and tumor cell vaccines.

**Results:**

Studies consistently showed that therapeutic vaccines, notably DC vaccines, had favorable safety profiles with few adverse effects. These vaccines, with varied antigenic formulations, demonstrated strong clinical outcomes, as indicated by metrics such as PSA response rates (9.5%‐58%), extended PSA doubling times (52.9%–89.7%), overall survival durations (17.7–33.8 months), two‐year mortality rates (0%–12.5%), biochemical relapse rates (42%–73%), and antigen‐specific immune responses (33.3%–71.4% in responsive groups).

**Conclusion:**

While clinical data for tumor vaccines have illuminated robust evidence of tumoricidal activity, the processes of their formulation and deployment are riddled with complexities. Combining vaccines with other therapies may enhance outcomes, and future research should focus on early interventions and deciphering the immune system's role in oncogenesis.

## 1 Introduction

**FIGURE 1:**
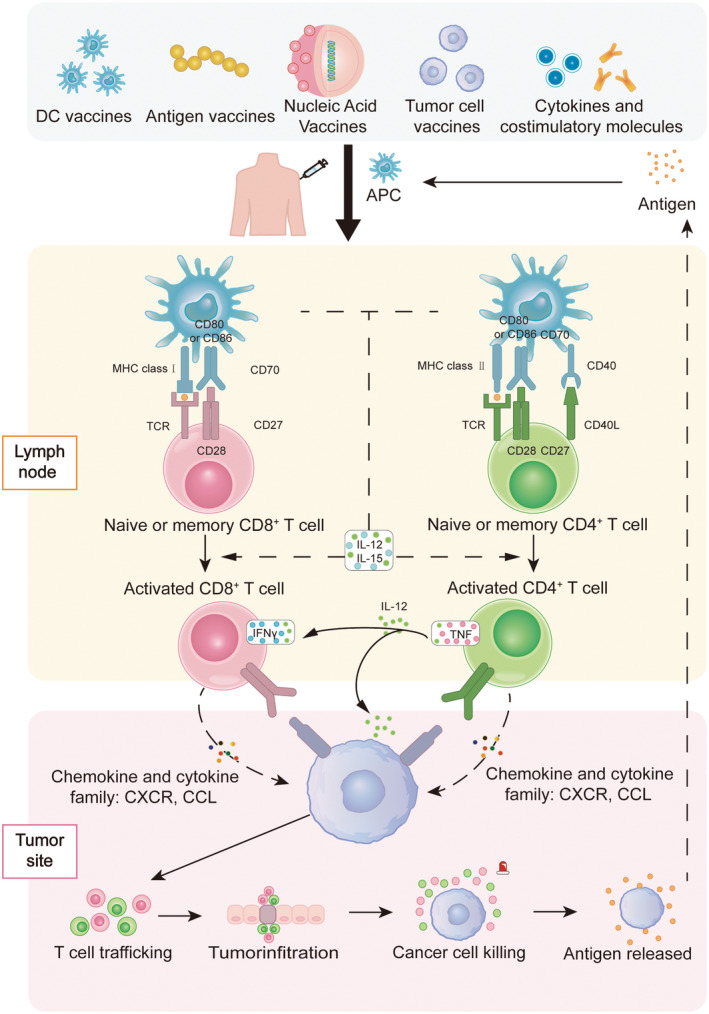
Tumor infiltration of therapeutic vaccines. Diverse therapeutic vaccines containing tumor antigens undergo processing by APCs upon entry into the body. These antigens are subsequently loaded onto MHC class I or MHC class II molecules on the APCs' surface within the fluid circulation, facilitating their transportation to lymphatic organs. Within these organs, APCs are stimulated to secrete IL‐12 and IL‐15, activating native or memory CD8^+^ and CD4^+^ T lymphocytes. This activation occurs through interactions between the TCR and homologous receptor‐ligand pairs. Simultaneously, CD4^+^ T lymphocytes release TNF and IL‐12, augmenting the anti‐tumor functions of CD8^+^ T lymphocytes. Ultimately, the activated T lymphocytes are mobilized around tumor cells via chemokines and cytokines, including the CXCR and CCL families, to facilitate the elimination of tumor cells. APC, antigen‐presenting cells; CCL, C‐C motif chemokine ligand; CXCR, C‐X‐C motif chemokine receptor; MHC, major histocompatibility complex; TCR, T‐cell receptor; TNF, tumor necrosis factor.

Across the globe, the incidence of prostate cancer (PCa) demonstrates an escalating trend annually. Notably in China, there is a steady rise in reported cases of PCa, accentuated by the recurring instances of biochemical recurrence (BCR) observed each year. This concerning pattern has garnered attention from the Chinese Government [1, 2, 3, 4]. PCa recognized as the most prevalent malignant epithelial tumor within the human urinary system, manifests a notable sensitivity to androgens during its initial stages of development. While early intervention through androgen‐targeted therapy yields favorable outcomes for patients encountering advanced PCa or those ineligible for surgical resection, its efficacy diminishes in the progression to castration‐resistant prostate cancer (CRPC). This transition renders traditional therapies, including chemotherapy, less effective, and prone to severe side effects, thus impeding advancements in treatment modalities. Tumor vaccines, a form of tumor‐specific active immunotherapy introduced in the 1990s, harness the body's immune system to sustain anti‐tumor responses, curbing recurrence and metastasis. Presently lacking standardized classification, most under‐development tumor vaccines are tailored to target specific tumor antigens. These therapeutic interventions aim at selective elimination of malignant cells while preserving normal tissue integrity. In principle, such vaccines possess the potential to impede the advancement of refractory tumors and advanced cancers resistant to established treatments such as surgery, radiotherapy, and chemotherapy. Their formulation involves the isolation, extraction, or synthesis of tumor‐specific or tumor‐related antigens in vitro [5]. The antigens are captured by antigen‐presenting cells (APCs), stimulating the body's T cells to generate specific cytotoxic T cells that bind and eliminate tumor cells (Figure 1). Early tumor vaccines involved infusing autologous or allogeneic tumor cells or combining them with non‐specific immune boosters like the Bacillus Calmette‐Guerin (BCG) vaccine and cytokines. However, antigens within the body struggle to be effectively presented and activate CTLs due to a lack of co‐stimulatory signals, challenges in overcoming immune tolerance, and the absence of distinct tumor‐associated antigens in most malignant tumors. Although the development and use of tumor vaccines face diverse challenges, ongoing research on tumor‐associated antigens, vaccine boosters, immune factors, host immunity, the tumor environment, and gene transfer technology has propelled many tumor vaccines into phase I, II, and III clinical trials. Progress in this field highlights the significant role tumor vaccines are expected to play in clinical tumor treatment [6, 7, 8, 9, 10, 11, 12]. Tumor vaccines encompass dendritic cell (DC)‐based, antigen (protein and peptide), nucleic acid (viral vector and DNA), tumor cell (autologous and allogeneic), and other vaccine variants [13, 14, 15, 16, 17]. Pharmaceutical and biotechnology companies have dedicated considerable efforts to tumor vaccine research and development, yet the creation of clinically potent tumor vaccines remains a formidable challenge. Consequently, extensive studies have assessed the efficacy of cancer vaccines, specifically those that harness self‐specific immune cells to target and eradicate cancerous cells. PCa exhibits distinct characteristics, notably featuring suitable target antigens, relatively diminished tumor burdens compared to other malignancies, and a comparatively slow rate of tumor progression. These distinctive traits pave the way for vaccines to elicit tailored immune responses, offering promising potential as impactful treatment avenues for PCa. To provide innovative insights into improved treatment strategies, we present a comprehensive analysis of the classification and current research status of therapeutic vaccines tailored for PCa within the domain of tumor immunotherapy.

## 2 Manuscript Content

### 2.1 Rationality of Immunotherapy in PCa

In contrast to the immediate impact of conventional chemotherapy on tumors, immunotherapy—encompassing antibody therapy, small molecule inhibitors, and vaccines—exhibits a more gradual and mild effect. Tumor vaccines necessitate the activation of the body's immune system to induce tumor regression, a process that takes a significant duration to become apparent. Consequently, immunotherapy emerges as particularly suitable for the treatment of slow‐growing tumors, exemplified by prostate and kidney cancers [18, 19, 20, 21, 22, 23, 24, 25].

Immunotherapy generally exhibits greater efficacy in tumors characterized by slow growth and smaller burdens, while its effects are less evident in tumors with larger diameters. This phenomenon may arise due to the suppression of immune microenvironment function by tumors with larger burdens. PCa typically presents a smaller tumor burden compared to other malignancies, rendering it more receptive to immunotherapy. Tumor antigens play a pivotal role in targeted immune therapy. Several tumor‐specific or associated antigens, including prostate‐specific antigen (PSA), prostate‐specific membrane antigen (PSMA), and prostate acid phosphatase (PAP), have demonstrated significant therapeutic potential in treating PCa. The existence of these antigens is imperative for vaccine‐based treatments for PCa. Reports have indicated the proliferation of T‐cell infiltrates within tumor tissues [26, 27, 28, 29, 30, 31, 32]. This occurrence is attributed to the stimulation of specific antigens. Moreover, the heightened presence of tumor‐infiltrating lymphocytes (TIL) correlates with favorable prognostic outcomes [33, 34]. Preclinical experiments have validated that clinical drug interventions and antitumor therapy can trigger immune responses in the prostate, leading to the infiltration of TILs into both PCa tissue and normal tissues [35, 36]. This evidence indicates that prostate tissue can instigate the infiltration of specific immune cells, creating a conducive environment for the effectiveness of therapeutic vaccines targeting prostate tumors.

### 2.2 Research Progress With Regard to PCa Vaccines

#### 2.2.1 Dendritic Cell‐Based Vaccines

DCs can promote immune response and the activation of anti‐tumor T cells through multiple mechanisms. In the tumor microenvironment, DCs can up‐take the tumor‐associated antigens which will be processed for further presentation. Meanwhile, they migrate to the peripheral immune organs, particularly lymph nodes, and mature. T cells can be recruited by the chemokines excreted by mature DCs. Furthermore, DCs can activate CD4^+^ T cells and CD 8^+^ T cells via the presentation of tumor antigens and MHC‐II and MHC‐I, respectively, and thus, initiate the tumor‐specific adaptive immune response [37]. The majority of DC vaccines derive from either DC cells or their mononuclear precursor cells sourced from patients, which are loaded with tumor antigens (known as DC/tumor fusion cells) in vitro and subsequently administered to the patients. The antigens represented by PSA are PCa‐associated and enable DCs to present specific antigens to T cells. These administered vaccines are capable of eliciting site‐specific anti‐tumor immune responses within the body. Within the immunosuppressive tumor microenvironment of PCa, where regulatory T cells and myeloid‐derived suppressor cells inhibit immune surveillance, DC vaccines aim to overcome these immune barriers by reactivating T‐cell responses, thereby strengthening the immune attack on cancer cells. Recently, DC immunotherapy has transitioned from basic in vitro vaccine cultivation to the development of targeted DC subcellular vaccines that focus on patient‐specific antigens.

Through ongoing technological advancements and refinement, novel DC vaccines have significantly contributed to the field of tumor immunotherapy. Continuous modifications and enhancements have led to noteworthy advancements in DC‐based therapy within tumor immunotherapy (Table 1) [38]. To date, the FDA has solely endorsed a single DC‐based vaccine, Sipuleucel‐T in 2010, for managing metastatic CRPC [39, 40]. Sipuleucel‐T utilizes autologous leukocytes obtained via the leukapheresis process from PCa patients. These leukocytes are co‐cultured with PCa antigens, thereby inducing the activation of immature DCs in vitro. Subsequently, the amplified and activated DCs are transfused back into the patient's body to stimulate T cells, thereby achieving anti‐tumor effects. The sensitized and matured DCs are then inoculated into patients with PCa. Sipuleucel‐T could improve the median survival of CRPC by 4.1 months compared with the placebo group (25.8 months vs. 21.7 months), with a hazard ratio of 0.78 (95% CI 0.61–0.98, *p* = 0.03), shown in phase III clinical trial [41]. Moreover, greater T‐cell proliferation responses were also observed in the Sipuleucel‐T group (73%), compared with the placebo (12.1%). Adverse events of chills, fever, headache, etc., were reported more frequently in the Sipuleucel T group, which was consistent with the release of cytokines. Generally, Sipuleucel‐T can significantly improve the overall survival (OS) of patients with CRPC. In a phase I/II clinical trial, Professor Podrazil combined the vaccine with docetaxel to treat 25 patients suffering from metastatic CRPC. Patients exhibited a median OS of 19 months, significantly surpassing the prognostic estimates of the Halabi and MSKCC nomograms (11.8 and 13 months, respectively). Additionally, there were no adverse reactions related to the vaccine. Kaplan–Meier survival analysis indicated a reduced risk of death for patients in comparison to the predicted values of the MSKCC and Halabi risk nomograms, concomitant with a significant reduction in peripheral blood regulatory T cells (Tregs). Prolonged vaccination can induce and sustain the proliferation of PSA‐specific T cells [42].

**TABLE 1:** Clinical trials involving DC vaccines.

Type or antigen	Authors	Population	Phase	Intervention	Clinical outcomes
MUC1	Scheid et al. [92]	nmCRPC	I/II	Arm 1: DCs charged with KLH‐DC (positive control for immune reactivity) Arm 2: Tumor MUC1 bearing Tn carbohydrates loaded DCs Arm 3: No protein as control	Safety: Adverse events: 94.0%; Clinical efficacy: PSA response: 0%; The mean PSADT:↑; PSA progression‐free: 12.5%; No radiologic progression: 25.0%; Mortality: 12.5%; CD4^+^ CD25^+^ Foxp3^+^: ↑*
PSA, MAGE‐1, and MAGE‐3	Jitka Fucikova et al. [93]	BCR of PCa	I/II	DCVAC/PCa vaccines	Safety: Adverse events: 3.7%; Clinical efficacy: The median PSADT: ↑; PSADT > 15 months: 68.0%; No difference: 12.0%. Mortality within 2 years: 0%; Metastases: 3.7%; CD3^+^: ↑(*p* < 0.05); Tregs (CD4^+^ CD25^+^ FoxP3^+^): ↓*
PSMA‐PA001	Guru Sonpavde et al. [46]	mCRPC	I	Arm 1: 1 × 10^6^ vaccine (I.D.) Arm 2: (1–5) × 10^6^ vaccine (I.D.) Arm 3: 1 × 10^6^ vaccine (I.V.) Arm 4: (1–5) × 10^6^ vaccine (I.V.)	Safety: Adverse events: 100.0%; Clinical efficacy: Total disease progression or died: 33.3%; OS: 17.7 months; Stable: 85.7%; PSA decrease: 35.3%; ≥ 25% increase in PSADT: 52.9%
hTERT, Survivin, PSMA1, STEAP1, and PAP	Tryggestad AMA et al. [85]	PCa at high risk of BCR	I/II	DC vaccine as adjuvant therapy after RALP	Safety: Well‐tolerated; Clinical efficacy: Total BCR‐free: 73%; ISUP 2–4 BCR‐free: 75%; ISUP 5 remained in BCR: 42%
NY‐ESO‐1, MAGE‐C2 and MUC1	Westdorp et al. [94]	CRPC	II	Arm 1: mDC vaccine Arm 2: pDC vaccine Arm 3: Combined vaccine (mDC + pDC)	Safety: Adverse events: 100.0% Clinical efficacy: DTH skin‐tests about NY‐ESO‐1‐specific CD8^+^: 71.4%; MAGE‐C2‐specific CD8^+^: 57.1%; MUC1‐specific CD8^+^: 23.8%; TAA‐specific responses: 71.4%; Median rPFS: 9.5 months; PSA↓: 9.5%
PSA; PSMA; surviving; Prostein; Trp‐p8	Fuessel et al. [95]	CRPC	I	DC Vaccination loaded with peptides derived from five antigens	Safety: Local skin reactions; Clinical efficacy: PSA↓: 12.5%; Antigen‐specific CD8^+^ activation: 37.5%
PSA; PSMA	Prue et al. [96]	CRPC	I	Arm 1: 1 × 10^6^ vaccine (I.D.) Arm 2: (1–5) × 10^6^vaccine (I.D.) Arm 3: 1 × 10^6^ vaccine (I.V.) Arm 4: (1–5) × 10^6^ vaccine (I.V.)	Safety: Adverse events: 66.7%; Clinical efficacy: DTH responses: 0%; KLH‐specific antibody responses: 33.3%; Peptide‐specific CD8^+^ responses and IFN‐γ production: 0%; PSA velocity: 0%

* *p* ≤ 0.05.

Abbreviations: BCR, biochemical relapse; CTCAE, common terminology criteria for adverse events; DC, dendritic cell; DTH, delayed hypersensitivity; HRQoL, health‐related quality of life; I.V., intravenous; I.D; mCRPC, metastatic castration‐resistant prostate cancer; mDC, myeloid DC; nmCRPC, non‐metastatic castration‐resistant prostate cancer; No., number; PCa, prostate cancer; pDCs, plasmacytoid DCs; PROMs, patient‐reported outcome measures; PSA, prostate‐specific antigen; PSADT, PSA doubling time; RALP, robot‐assisted laparoscopic prostatectomy; rPFS, radiological progression‐free survival; trp‐p8, transient receptor potential p8.

#### 2.2.2 Antigen Vaccines

Antigen vaccines primarily consist of one or several proteins or peptides acting as antigens. These vaccines utilize tumor‐specific antigens (referred to as protein fragments or peptides), such as PSA, PAP, and PSMA in PCa, to trigger the immune system, promoting the production of targeted antibodies or cytotoxic T lymphocytes (also known as killer T cells) for the destruction of tumor cells bearing these specific antigens. Basically, these tumor‐specific antigens are presented by MHC‐I and MHC‐II molecules on APCs to B and T lymphocytes, triggering humoral and cellular immunity that precisely targets tumor cells. The activation of both tumor‐specific CD4^+^ and CD8^+^ T cells enhances the anti‐tumor effects of the body [43]. To ensure that vaccination‐induced immune responses selectively target tumor cells carrying the antigen rather than normal cells, the antigens utilized in tumor vaccines differ from those presented by normal cellular molecules [44]. The antigen‐based vaccines can selectively target overexpressed or PSAs, enhancing the immune system's ability to recognize and attack tumor cells.

In a phase I/II clinical trial, Professor Wolfgang Lilleby employed a novel hTERT peptide vaccine named UV1 to treat 22 patients diagnosed with confirmed adenocarcinomas of the prostate and non‐visceral spread [45]. Immunological responses to UV1 peptides were confirmed in 85.7% of the patients, with PSA levels dropping to < 0.5 ng/mL in 64% of them, and MRI scans showing no evidence of persistent tumor in the prostatic gland in 45% of the patients. By the end of the nine‐month reporting period, 77.2% of the patients exhibited clinically stable disease confirmed by PSA < 0.2 ng/mL and/or MRI evaluation. The UV1 peptide vaccine treatment showed a minimal occurrence of adverse events, most of which were injection site pruritus and pollakiuria, with no dose‐limiting toxicities and induced specific immune responses in a significant proportion of patients, irrespective of their HLA type. In phase I clinical trial, Professor Guru Sonpavde administered the antigen vaccine known as BPX101 to 18 patients with advanced PCa [46]. Approximately 33.3% of the patients faced disease progression or mortality, exhibiting a mean progression‐free survival (PFS) of 269.5 days and a mean OS of 477 days. Grade 1 adverse events were mostly observed encompassing injection site erythema, fatigue, injection site induration, etc. A total of seven grade 3 adverse events were considered unrelated or unlikely associated with the treatment. However, the assessment of the clinical efficacy was limited by its sample size and the stage of the trial, necessitating further research. In general, these outcomes suggest enhanced safety, immune response, and anti‐tumor activities in both chemo‐naive and post‐chemotherapeutic patients with advanced PCa.

#### 2.2.3 Nucleic Acid Vaccines

Nucleic acid vaccines are categorized into viral vector, DNA, and RNA vaccines, offering the combined benefits of live attenuated and recombinant viral vector vaccines. Additionally, they mitigate safety challenges associated with the preparation of live attenuated vaccines [47]. Viral vaccines exhibit natural immunogenicity upon entry into the human body, allowing the design of vaccines containing specific tumor antigens based on the virus's genetic sequence. Typically, adenoviruses serve as vectors to infect immune cells, notably APCs like DCs, triggering T‐cell activation through viral vaccines upon entering the body, resulting in the production of anti‐tumor immunity. Additionally, oncolytic viruses can serve as carriers, lysing tumors themselves, releasing tumor antigens, and enhancing vaccine effectiveness to induce long‐term immune memory [48]. Studies related to nucleic acid vaccines, particularly DNA and viral vector vaccines, have demonstrated their safety and reliability. RNA vaccines facilitate the release of tumor‐derived vaccines and specific antigens, inducing both humoral and cellular immune responses while offering improved tolerance through co‐stimulatory signals [49, 50].

Nucleic acid vaccines can target PCa due to their ability to encode PSAs, such as PSA, PAP, and PSMA. These antigens are highly expressed in PCa cells, enabling the vaccine to induce a specific immune response that targets and eliminates cancer cells, stimulating a T‐cell‐mediated attack against the tumor. The immune response in the tumor microenvironment of PCa can be activated by vaccines via multiple pathways. For instance, DNA vaccines trigger innate immunity through pathways like TLR9, AIM2, and STING, promoting cytotoxic T‐cell responses. These pathways help overcome the immunosuppressive tumor microenvironment, enabling effective immune cell infiltration and tumor cell destruction [51].

DNA vaccines employ plasmid DNA encoding specific antigen sequences and are administered via intramuscular or subcutaneous injection. Antigens are processed and taken up by APCs, which subsequently present to both CD8^+^ and CD4^+^ T cells via MHC class I and II molecules, respectively, eliciting specific humoral and cellular immune responses [52, 53].

RNA has been utilized in cancer treatment research for an extended duration [54]. In comparison to DNA vaccines, RNA vaccines offer enhanced safety and degradation ease and do not integrate into the host's genome [55]. Additionally, DNA vaccines require transfection into the nucleus for efficacy, whereas RNA vaccines solely need to access the cytoplasm. In contrast to protein vaccines, RNA vaccines swiftly evoke diverse immune responses [56]. The instability of RNA restricts its vaccine applications, resulting in a heavy reliance on DCs for developing PCa RNA vaccines. Studies have confirmed the efficacy of PCa RNA vaccines based on DCs. Herser et al. [57] transfected DCs with PSA RNA for PCa treatment, inducing strong CTL immune responses in patients without adverse effects. Additionally, immature DCs can ingest and process RNA vaccines, facilitating their transition to semi‐mature states. Su et al. [58] utilized hTERT mRNA‐transfected DCs to treat advanced PCa, eliciting immune responses from antigen‐specific CD8^+^ and CD4^+^ T cells.

Despite the absence of approved clinical nucleic acid vaccines, RNA vaccines are well‐tolerated and capable of releasing tumor‐specific antigens to trigger immune responses without carcinogenic potential [59].

#### 2.2.4 Tumor Cell Vaccines

A tumor cell vaccine is a therapeutic formulation comprising numerous antigens derived from tumor cells. These cells are commonly deactivated through radiation in laboratory settings. Despite similar principles, tumor cell vaccines own stronger immunogenicity due to their carrying more tumor‐associated antigens compared with antigen‐based vaccines. However, the immune escape mediated by antigen modulation, up‐regulated expression of inhibitory factors like CTLA‐4 and toll‐like receptors, and the generation of immunosuppressive environments of tumor cells has greatly limited the immunogenicity of tumor cells [60, 61]. So, typically, tumor cell antigenicity is heightened by incorporating chemical components or introducing new genes before inoculation into patients. The patient's immune system identifies antigens on the surfaces of these tumor cells, selectively targeting cells expressing matching antigens. These vaccines target PCa by utilizing the naturally expressed tumor‐specific antigens, such as PSA, PAP, and PSMA, present in PCa cells, with or without additional genetic modifications.

Tumor cell vaccines are categorized as autologous or allogeneic based on their origin [62, 63, 64, 65]. Autologous tumor cell vaccines involve extracting tumor cells from patients' tissues undergoing treatment, rendering them non‐tumorigenic while preserving their immunogenic properties. These vaccines can be administered post‐surgery or preserved through culturing or freezing for future applications. Allogeneic tumor cell vaccines, derived from other patients rather than the treated individual's cells, resemble personalized medicines more than traditional vaccines. Both autologous and allogeneic tumor cell vaccines share one similar mechanism of facilitating immune responses that they can increase the production of IL‐2 and facilitate Th1 immunity, leading to the augmentation of downstream CD8^+^ and macrophages' responses [43, 66].

Occasionally, primitive tumor cell vaccines fail to elicit robust immune responses. To address this limitation, current tumor vaccines undergo modification via co‐stimulatory or MHC molecules, altering the immune characteristics or genetic profiles of tumor cells to enhance their immunogenicity and prompt stronger immune responses. Yet, inherent limitations like poor immunogenicity and the risk of tumorigenesis have led to the replacement or enhancement of most traditional tumor cell vaccines with novel therapies like DC therapy and Chimeric Antigen Receptor T‐Cell (CAR‐T) immunotherapy, among others. Unlike the DC vaccine, CAR‐T represents a relatively recent form of tumor immunotherapy rather than fitting within the conventional vaccine category.

#### 2.2.5 Viral Vector Vaccines

Viral vector vaccines utilize the ability of delivering genes of the virus to achieve anti‐tumor effects by integrating genes that express tumor antigens or other tumor‐suppressive genes into the genomes of body cells, leading to the expression of specific proteins. Adeno‐associated viruses and herpes simplex virus are the most widely used vectors at present [67]. Virus‐based vaccines enhance specific immunity through direct presentation and cross‐presentation mechanisms. Direct presentation involves the vaccine directly stimulating APCs to synthesize encoded proteins and present them in MHC environments. Cross‐presentation occurs when the virus infects different cell types, such as epithelial cells, leading to the lysis of adjacent APCs to uptake cell debris and present synthesized proteins on MHCs. Both methods result in inducing immunity against encoded proteins. By delivering PCa‐associated antigens, such as PSA, into host cells, viral vector‐based vaccines can prompt these cells to express tumor antigens, stimulating both cellular and humoral immune responses.

### 2.3 Cytokines and Co‐Stimulatory Molecules That Improve T‐Mediated Anti‐Tumor Immunity

Presently, the clinical utilization of numerous vaccines relies significantly on the synergistic impact of cytokines and co‐stimulatory molecules [68, 69]. These molecules promote APC proliferation, maturation, and activities, inducing high‐affinity CD4^+^ and CD8^+^ T‐cell receptors, thereby enhancing immune responses. Table 2 provides a concise overview of the cytokines and combination therapies utilized in current vaccine applications. GM‐CSF, in comparison to other cytokines, significantly activates cellular immune responses. GM‐CSF serves as a crucial adjuvant for tumor immunity, demonstrating significant anti‐tumor effects and offering several advantages in combination with chemotherapy and targeted therapy. GM‐CSF promotes APC proliferation and maturation, augments the expression of MHC molecules, facilitates antigen presentation by APCs, and boosts their vitality [70, 71, 72]. GM‐CSF and IL‐12 synergistically stimulate CD8^+^ T‐cell‐mediated immune responses [73, 74]. Furthermore, many clinical applications involve tumor cell lines transduced with GM‐CSF or co‐cultivated with GM‐CSF to yield improved outcomes.

**TABLE 2:** Clinical trials of cytokines or combination therapy.

Content	Authors	Population	Phase	Intervention	Clinical outcomes
GM‐CSF	Rini et al. [97]	SPPC	II	250 μg/m^2^/day of GM‐CSF	Safety: Adverse events: 82.8%; Clinical efficacy: PSA↓: 10.3%; PSADT↑: 89.7% ***; The median slope of the PSA versus time curve↓: 89.7%**
GM‐CSF; ipilimumab	Van den Eertwegh et al. [89]	mCRPC	I	Arm 1: GVAX +0.3 mg/kg ipilimumab Arm 2: GVAX +1.0 mg/kg ipilimumab Arm 3: GVAX +3.0 mg/kg ipilimumab Arm 4: GVAX +5.0 mg/kg ipilimumab	Safety: Adverse events: 71.4%; Clinical efficacy: PSA↓: 25.0%; Immune reactivity about filamin B positive: 46.4%; median OS: 33.8 months; Immune reactivity about PSMA positive: 42.8%
PGE‐2; TNF‐α	Thomas‐Kaskel et al. [90]	CRPC	I/II	Arm 1: PSCA + PSA peptides Arm 2: CPP‐PSCA + PSA peptides	Safety: Toxicity events: 0%; Clinical efficacy: DTH to tumor peptide: 50.0%; Stable disease: 40.0%
Cytokine cocktail	Mu et al. [98]	CRPC	I/II	Arm 1: I.N. Arm 2: I.D.	Safety: Toxicity events: 0%; Clinical efficacy: PSA↓: 68.4%; Immunological response about ELISPOT: 52.6%
Cytokine cocktail	Reyes et al. [99]	CRPC	I	—	Safety: Adverse events: 45.0%; Clinical efficacy: PSA↓: 42.9%; Immunological response about ELISPOT: 50.0%; Immunological response about DTH: 64.3%
TNF‐α, PGE‐2	Frank et al. [100]	CRPC	I	Arm1: Vaccine on Week 1–7 Arm 2: Placebo on Week 1–7, vaccine on Week 8–14	Safety: Adverse events: 91.7%; Clinical efficacy: Immunological responses about DTH: 66.7%; PSADT differences between two groups**
Cytokine cocktail	Kongsted et al. [91]	CRPC	II	Arm 1: Docetaxel Arm 2: Docetaxel + DC vaccine	Safety: Local reactions and rash; Clinical efficacy: PSA↓: 58% vs. 38%; PFS: 5.5 vs. 5.7 months

** *p* ≤ 0.01.

*** *p* ≤ 0.001.

Abbreviations: CPP, cell‐penetrating peptide; DTH, delayed hypersensitivity; ELISPOT, enzyme‐linked immunospot technology; GM‐CSF, granulocyte‐macrophage colony‐stimulating factor; GVAX, the granulocyte‐macrophage colony‐stimulating factor‐transduced allogeneic prostate cancer cells vaccine; I.D., intradermal; I.N., intranodal; mCRPC, metastatic castration‐resistant prostate cancer; PFS, progression‐free survival; PSADT, PSA doubling time; PSMA, prostate‐specific membrane antigen; SPPC, serologic progression of prostate cancer.

The affinity between TCR and target antigens significantly influences CD8^+^ cell‐specific immune responses. Yet, low‐concentration antigens in vivo pose challenges in inducing high‐affinity CD8^+^ T cells, often leading to immune tolerance. Consequently, concurrent transduction of costimulatory molecules, like GM‐CSF and TNF‐α, assists in inducing high‐affinity CD8^+^ T cells and enhancing cellular immune responses [75].

IL‐15, a cytokine belonging to the chemokine family, exhibits diverse immunomodulatory functions. It participates in signaling and activating T cells, B cells, and NK cells, contributing to their proliferation and survival [76]. IL‐15 activates and expands CD8+ memory T cells, promoting cell proliferation, and activation, and boosting IFN‐γ and TNF‐α secretions. Additionally, it stimulates the expression of co‐stimulatory factors and IFN‐γ by DCs, enhancing DC cells' ability to activate CD8^+^ T and NK cells [77]. IL‐15 significantly contributes to the proliferation and activation of NK cells. IL‐15 gene knockout in mice led to varying reductions in NK cell abundance, whereas overexpression of IL‐15 significantly increased NK cell levels along with improved immune responses [78]. Studies involving animal experiments and clinical trials have investigated the therapeutic potential of IL‐15 in disease treatment.

Additionally, various cytokines including IL‐2, IFN‐γ, and IL‐12, among others, stimulate immune cell proliferation and activity, augmenting the body's cellular immune responses.

### 2.4 Challenges and Prospects of Clinical Applications of PCa Tumor Vaccines

While certain initial clinical trial data on tumor vaccines demonstrated significant evidence of immunogenicity and targeted killing of tumor cells, additional vaccines proved less effective in enhancing both quality of life and survival advantage [79]. Presently, numerous vaccine clinical trials employ OS as their primary endpoint. However, OS necessitates a prolonged follow‐up period, introducing confounding factors from other combined treatments that bias efficacy assessments. Although PSA and its doubling time (PSADT) serve as crucial prognostic indicators for PCa, they inadequately capture the disease's dynamics. Hence, there exists a necessity to formulate evaluation systems for assessing the clinical efficacy of vaccine treatments. Likewise, tumor vaccines, as biologically active macromolecular antigens, comprise a range of antigenic components, including whole‐cell lysates used for antigen provision. When combined with adjuvants, vaccine components become more complex, differentiating vaccine preparation from that of traditional antitumor drugs. Consequently, during clinical trials, substantial variations in efficiency arise due to the complexity of vaccine components among individuals receiving the same vaccine regimen and trial treatment. Ultimately, patients and researchers may reject vaccines with similar biological mechanisms due to the poor efficacy of a single vaccine. Consequently, numerous scholars [80, 81, 82, 83] advocate for personalized tumor vaccine preparation. However, the rising costs associated with this individualized approach pose challenges for subsequent multi‐center clinical trials. Despite extensive studies on PCa, the precise biological mechanisms and tumor immune microenvironment contributing to PCa's onset and progression, particularly in the case of CRPC, remain undetermined. Thus, focusing research efforts solely on understanding and addressing the tumor immune microenvironment and molecular mechanisms is crucial for enhancing the efficacy of tumor vaccines. Given that tumor vaccine treatment remains a nascent field in clinical practice, many researchers in the clinical domain struggle to grasp optimal intervention strategies involving combinations of immunotherapy. Additionally, there is a dearth of sufficient screening criteria for patients participating in diverse clinical trials. Variations in assessing efficacy and adverse reactions result in inconsistent vaccine applications, consequently impacting the trial procedures.

In summary, beyond the aforementioned challenges, enhancing the induction of T‐cell responses, particularly maximizing the activation and expansion of CD8^+^ T cells, and identifying suitable vaccine delivery systems are imperative. This would enable swift, cost‐effective production and timely deployment of vaccines. Although personalized therapeutic vaccines may offer substantial clinical benefits to cancer patients, their preparation and application pose numerous challenges. Considering the complexities inherent in developing therapeutic vaccines for tumors, there is a pressing need for a comprehensive comprehension of the immune system's role in cancer inception. This includes addressing the formidable challenges linked to assessing treatment responses and devising research and development strategies commensurate with the vast research prospects.

## 3 Discussion and Conclusions

Despite over a century of exploratory studies on tumor vaccines, developing them into clinically effective therapies remains challenging. PCa is notably responsive to immunotherapy, attracting significant scholarly attention in PCa tumor vaccine research. Some vaccines have progressed to clinical trials, demonstrating the ability to trigger antigen‐specific immune responses with improved safety profiles regarding short‐ and long‐term side effects [84, 85, 86]. Nevertheless, the composition of PCa vaccines is intricate, and the advantages and disadvantages of target antigens remain ambiguous. Establishing an evaluation system for PCa target antigens and consistently screening for optimal tumor vaccine targets is crucial.

While DC tumor vaccines exhibit personalized treatment features targeting the PSMA, known for their high specificity and membrane binding abilities [87], the procedures for acquiring DCs, selecting tumor antigens, and devising treatment plans significantly influence the therapeutic outcomes of these vaccines. Despite the promising application prospects of DC tumor vaccines, several unresolved challenges persist, such as uncertainty regarding tumor antigens, heterogeneity among DC subgroups, and their limited presentation capabilities. Currently, in our country, both medical technology and regulatory policies regarding DC vaccines require further advancements, particularly in the standardized preparation of these vaccines. For instance, regarding oncology selection and antigenic determination, despite the growing number of studies on DC tumor vaccines, most clinical trials have utilized DC sensitization with lysates derived from patient tumor tissues. The uncertainty surrounding tumor antigens has led to ambiguous clinical outcomes for these DC vaccines due to the absence of well‐defined tumor‐associated or tumor‐specific antigens. Owing to safety and efficacy concerns associated with DC vaccines derived from peripheral blood, patients' autologous DCs remain the primary DCs used in clinical research. Nevertheless, current in vitro expansion methods can yield only a restricted number of DCs, necessitating frequent collections of peripheral blood from patients. Consequently, research on in vitro expansion systems should be conducted to determine the cell quantity meeting the treatment necessities.

The ongoing advancements in genomics and molecular biology have led to the utilization of an increasing array of tumor antigens in clinical research. Tumor cells can be genetically engineered to express pro‐inflammatory cytokines, such as GM‐CSF, to boost immune responses. This method has been tested in various clinical trials, particularly in PCa vaccines, showing potential. For example, the GVAX vaccine is a genetically modified tumor cell vaccine that can express human *GM‐CSF* gene, which aims to stimulate DCs for stronger immune responses [73, 74]. Challenges confronting antigen vaccines involve crafting personalized vaccines and encountering delays in the manufacturing process. Identifying individualized tumor antigens necessitates intricate patient‐specific detection and analysis, a highly complex and time‐intensive procedure. Moreover, improving manufacturing processes is essential for ensuring the consistency of individualized tumor antigens during their preparation. Hence, there's an urgent need for a broader spectrum of advanced technologies. It is anticipated that future advancements will significantly reduce both the time and production costs involved in this process.

The mechanisms of nucleic acid vaccines and viral vector vaccines share certain similarities, as both involve the transfection of cells with DNA or RNA to express tumor‐specific antigens, thereby inducing an immune response. However, the delivery of DNA vaccines remains a technical challenge; plasmids are commonly used as a delivery means, but it is difficult for plasmids alone to pass through the nuclear membrane and reach the cell nucleus for expression. To address this, Professor Yachnin employed electroporation to enhance delivery efficiency and increase cellular uptake of DNA, thereby improving the immunogenicity of the vaccine [88]. Another way for transfecting cells with DNA vaccines involves using viral vectors, which can actively integrate into the host cell genome to express specific proteins. On the other hand, mRNA vaccines can be directly translated into the cytoplasm without the need for integration into the host genome, offering higher safety and overcoming some limitations of DNA vaccines. Due to these advantages, mRNA vaccines have garnered increasing attention, though they are still in the early stages of development and require further research. Combining vaccines with other therapeutic approaches, such as co‐administration with other type of vaccines, immunotherapy, or chemotherapy, is also being explored as a means to enhance efficacy [89, 90, 91]. This approach offers new directions and opportunities for the clinical application of vaccines.

**FIGURE 2:**
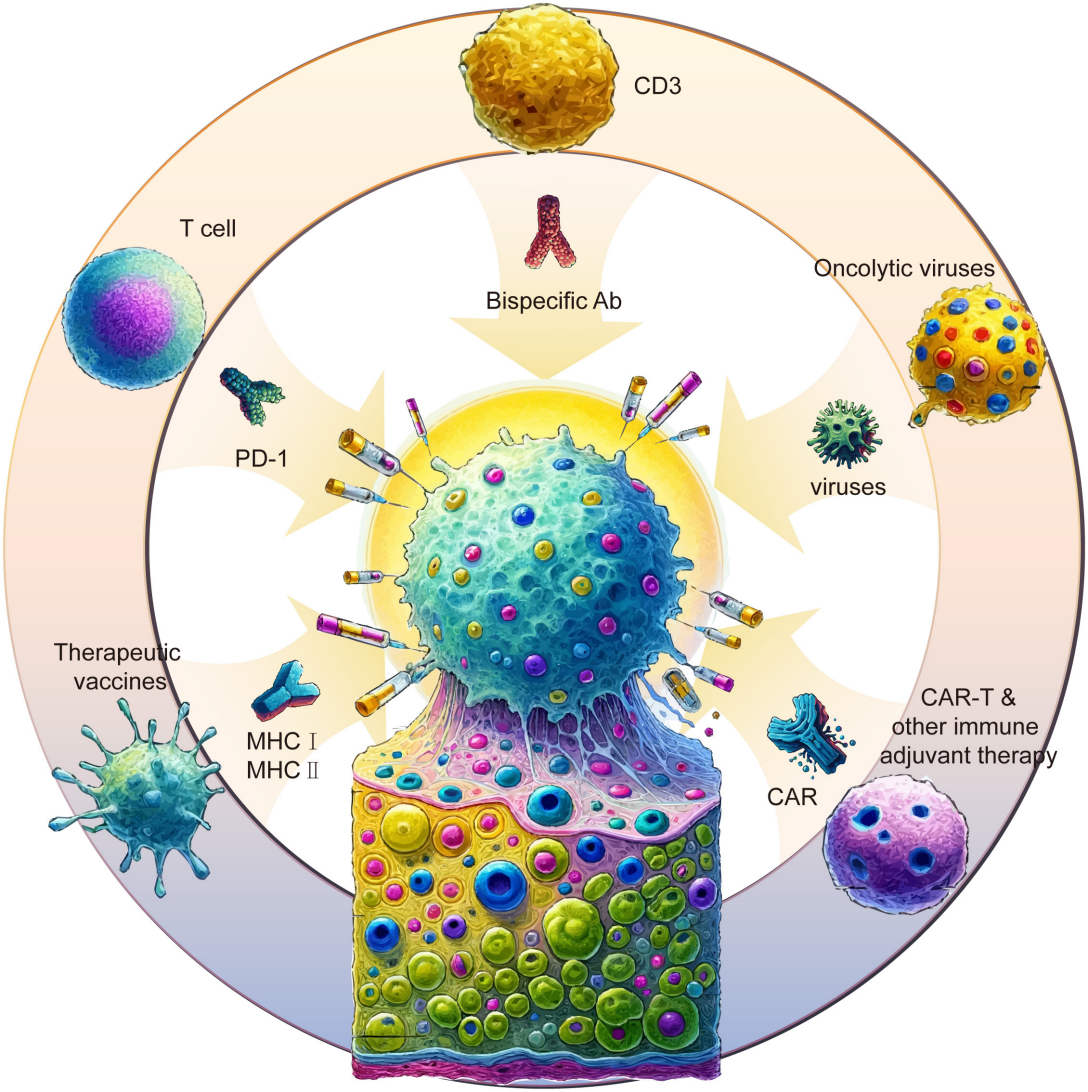
Combination of immunotherapies in prostate cancer. Comprehensive immunotherapeutic approaches to eradicate prostate tumor cells encompass therapeutic vaccines formulated with tumor antigen proteins or peptides, along with viral vectors carrying antigen genes. In vivo, these antigen‐loaded vaccines stimulate APC functions, bolster DC activity, modulate CD4^+^ T cells to regulate T regulatory cell functions, and enhance the anti‐tumor capabilities of CD8^+^ T cells. Additionally, CAR‐T therapy and other immune adjuvant therapies can deploy tumor‐specific CD8^+^ T cells directly infused into the body to achieve anti‐tumor efficacy. Furthermore, immune checkpoint inhibitors possess the capacity to obstruct inhibitory pathways like PD‐1/PD‐L1 and CTLA‐4 in tumor microenvironments, countering the suppression of T‐cell functions and reinforcing anti‐tumor efficiency. Oncolytic viruses, engineered to selectively replicate and eliminate tumor cells, augment the immunotherapeutic methods' ability to efficiently present tumor antigens to the immune system. These viruses can function as stand‐alone therapies or in combination with other immunotherapeutic methods. The synergistic use of these methods facilitates the generation and activation of tumor‐specific T cells, culminating in a collaborative killing effect, offering a therapeutic strategy to counteract existing immunotherapy challenges. APC, antigen‐presenting cells; CAR‐T therapy, chimeric antigen receptor T‐cell immunotherapy; CTLA‐4, cytotoxic T‐lymphocyte‐associated protein 4; DC, dendritic cell; PD‐1/PD‐L1, programmed death 1/programmed death ligand 1.

To summarize, despite the promise of vaccine therapy in clinical practice, numerous hurdles must be overcome for its practical application, necessitating extensive basic and clinical research for improvement. Additionally, the vaccine's independent therapeutic effects are unsatisfactory. Consequently, multiple studies have attempted to augment its efficacy by combining it with other treatment modalities such as oncolytic viruses, CAR‐T, immune checkpoint inhibitors, and other immune adjuvant therapy strategies (Figure 2). These combined applications demonstrate good tolerance, a crucial aspect for therapeutic effectiveness. Future studies on PCa vaccines will likely focus on patients receiving earlier treatments, having smaller tumor burdens, and possessing better prognostic factors. Additionally, establishing the optimal timing for early interventions is crucial to augment the outcomes of tumor vaccine treatments.

## Author Contributions

**Jide He:** data curation (equal), formal analysis (lead), writing – original draft (lead). **Jialong Wu:** data curation (equal), formal analysis (equal), writing – original draft (equal). **Ziang Li:** data curation (supporting). **Zhenkun Zhao:** data curation (supporting). **Lei Qiu:** data curation (supporting). **Xuehua Zhu:** data curation (supporting). **Zenan Liu:** data curation (supporting). **Haizhui Xia:** formal analysis (equal). **Peng Hong:** formal analysis (equal). **Jianling Yang:** formal analysis (equal). **Ling Ni:** conceptualization (equal), project administration (equal), resources (equal), supervision (equal), writing – review and editing (equal). **Jian Lu:** conceptualization (equal), funding acquisition (lead), project administration (equal), resources (equal), supervision (equal), writing – review and editing (equal).

## Ethics Statement

The authors have nothing to report.

## Consent

The authors have nothing to report.

## Conflicts of Interest

The authors declare no conflicts of interest.

## Data Availability

Data sharing not applicable to this article as no datasets were generated or analyzed during the current study.
